# The Central Role of Non-Structural Protein 1 (NS1) in Influenza Biology and Infection

**DOI:** 10.3390/ijms21041511

**Published:** 2020-02-22

**Authors:** Nícia Rosário-Ferreira, António J. Preto, Rita Melo, Irina S. Moreira, Rui M. M. Brito

**Affiliations:** 1 Coimbra Chemistry Center, Chemistry Department, Faculty of Science and Technology, University of Coimbra, 3004-535 Coimbra, Portugal; 2 CNC—Center for Neuroscience and Cell Biology. University of Coimbra, UC Biotech Building, 3060-197 Cantanhede, Portugal; 3 Centro de Ciências e Tecnologias Nucleares and Departamento de Engenharia e Ciências Nucleares, Instituto Superior Técnico, Universidade de Lisboa, 2695-066 Bobadela LRS, Portugal; 4 Department of Life Sciences, University of Coimbra, 3000-456 Coimbra, Portugal

**Keywords:** protein structure, structural bioinformatics, molecular modeling, protein–protein interactions, PPIs, influenza virus, NS1

## Abstract

Influenza (flu) is a contagious viral disease, which targets the human respiratory tract and spreads throughout the world each year. Every year, influenza infects around 10% of the world population and between 290,000 and 650,000 people die from it according to the World Health Organization (WHO). Influenza viruses belong to the Orthomyxoviridae family and have a negative sense eight-segment single-stranded RNA genome that encodes 11 different proteins. The only control over influenza seasonal epidemic outbreaks around the world are vaccines, annually updated according to viral strains in circulation, but, because of high rates of mutation and recurrent genetic assortment, new viral strains of influenza are constantly emerging, increasing the likelihood of pandemics. Vaccination effectiveness is limited, calling for new preventive and therapeutic approaches and a better understanding of the virus–host interactions. In particular, grasping the role of influenza non-structural protein 1 (NS1) and related known interactions in the host cell is pivotal to better understand the mechanisms of virus infection and replication, and thus propose more effective antiviral approaches. In this review, we assess the structure of NS1, its dynamics, and multiple functions and interactions, to highlight the central role of this protein in viral biology and its potential use as an effective therapeutic target to tackle seasonal and pandemic influenza.

## 1. Introduction

Influenza viruses can infect a vast array of animal hosts. They feature an evolutionary ability to explore genetic diversity through gene changes such as antigenic shift or antigenic drift, boosting flu’s seasonal or pandemic outbreaks. Influenza infects over one billion people worldwide each year with three to five million of them affected by severe symptoms and around 290 to 650 thousand deaths each year [1,2]. Worldwide statistics show that over three million dollars are spent every year in influenza-related hospital care [3]. Thus, the worldwide annual financial impact of seasonal influenza is enormous, but pales in comparison with a pandemic outbreak of influenza which can go up to three trillion dollars [4]. For example, during the most recent worldwide outbreaks of H5N1 (Asian flu or bird flu) and H1N1 (swine flu), governments of affected countries spent in excess of 80 billion dollars [5]. In addition, influenza is often misdiagnosed making it difficult to accurately assess the financial impact of the disease [6].

Healthy adults are not as susceptible to serious complications as at-risk populations, the oldest, the youngest, people with respiratory pathologies or compromised immune system. In most cases, symptoms are mild and nonspecific such as fever, cough, rhinorrhea, and myalgia. In the most complicated cases, the prescription of antivirals is recommended. Pandemic outbreaks occur occasionally and abruptly, and therefore healthy adults are also at risk. To control flu outbreaks, extreme actions such as restricting traveling or access to public places have been implemented which have a significant impact on the global economy [7,8].

Influenza viruses are divided into different subtypes depending on the antigenic properties of two proteins on their surface, hemagglutinin (HA) and neuraminidase (NA). In principle, it is possible to have combinations of any of the 18 hemagglutinins with 11 described neuraminidases. However, to date, not all have been detected in animals or humans. The diversity of virus subtypes is a major hurdle for vaccine effectiveness as vaccine production takes into account the strains circulating each season, each year [9].

Mutation rates in the influenza virus are high rendering insufficient previously acquired host immunity. The antigenic changes result from two different processes, i.e., antigenic drift and antigenic shift [7]. Antigenic drift results from subtle changes in the influenza virus’ genome over time that culminates in a virus of the same strain, but unrecognizable for the host cell immune system. These mutation high rates occur because of the lack of proofreading ability in RNA viruses and it is common in several of these viruses. Unlike antigenic drift, common in several viruses, influenza A viruses (IAV) display a rare ability to undergo antigenic shift [10]. This process, also known as reassortment, occurs occasionally when segments of RNAs from two different strains of IAV infect the same cell allowing their rearrangement into a new viral strain for which most of the population does not have preexisting immunity. A new viral strain can arise due to this genetic event which may display the ability to spread successfully from human-to-human, allowing a new pandemic [11]. 

In the last one hundred years, the Centers for Disease Control and Prevention (CDC) records have registered four influenza pandemics [12]: 1918, 1957, 1968, and 2009. In the 1918 pandemic, known as the Spanish flu, over one-third of the world’s population became infected and 50 million people died, making this the largest influenza pandemic in terms of mortality. Its origin may be traced to the reassortment of a human H1 and an avian N1 viral subtypes. The 1957 pandemic (Asian flu) caused over 1.1 million deaths from infection by an H2N2 viral subtype which resulted from the combination of the H1N1 from the 1918 pandemic with an avian H2N2 subtype. The Asian flu subtype then reassorted with the H3 avian subtype originating the H3N2, responsible for the 1968 pandemic causing over one million victims. The latest pandemic, registered in 2009, resulted from the reassortment of the human H3N2, the swine H1N1, and the avian H1N2 subtypes. This pandemic infected over 60 million people in the United States, killing from 100 to 500 thousand people worldwide [13,14,15]. The threat of a new pandemic is always present, as shown by the recent situation in China with a H7N9 Asian flu epidemic that was contained after the use of the H7-Re1 vaccine. However, the virus underwent antigenic shift and a new highly pathogenic avian influenza (HPAI) virus was identified last year [16]. 

To reduce influenza’s infections three main approaches are available: vaccination, antiviral drugs, and surveillance. Vaccination is the main preventive response to influenza infections. As mentioned before, vaccines get annual updates according to information from WHO’s Global Influenza Surveillance and Response System (GISRS), responsible for data collection and analysis on surveillance, clinical practice, and research around the world [11,13]. When vaccination fails, antiviral drugs are the next alternative. The available antivirals inhibit either the viral matrix protein 2 (M2) [17] (amantadine and rimantadine) or neuraminidases [17] (oseltamivir, zanamivir, laninamivir and peramivir). However, CDC does not recommend the use of the first two for recently circulating influenza viruses due to the high resistance they display towards these drugs; hence neuraminidase inhibitors are the only used in-clinic currently [18]. Usually, these drugs are administered as monotherapy and fall short of their therapeutic outcome as they are relatively low in efficacy against seasonal or pandemic flu, only lowering the infection rate [19]. These limitations enhance the need to develop new therapeutic approaches through the knowledge of virus biology and host interactions [20]. Several efforts have been reported to develop new approaches, like monoclonal antibodies, against known targets such as neuraminidase [21] or to identify alternative targets like the nucleoprotein [22] and the polymerase acidic protein (Baloxavir Marboxil) [23]. 

In this review, we focus on influenza’s Non-Structural Protein 1 (NS1). NS1 presents itself as a potential therapeutic target based on two central complementary activities, i.e., counteracting the host’s immune response and aiding viral replication [24]. Previous studies on the role of NS1 in the virus biology have shown that viruses without this protein have low rates of replication and dissemination. Thus, the development of therapeutic approaches targeting NS1 could achieve good outcomes in influenza infection treatment [25,26,27].

## 2. The Influenza Virus

The influenza virus belongs to the *Orthomyxoviridae* family of virus. There are four types (A, B, C and D) of influenza virus but only A, B, and C infect humans. Human influenza A and B are the virus types responsible for the seasonal flu epidemics, whereas influenza type C infections generally cause mild illness. Influenza A viruses are the only influenza viruses known to cause flu pandemics. The influenza viruses from types A and B are pleomorphic with size ranging from 80 to 120 nm. Their shape is typically spherical but can acquire a cordlike shape. The C-type influenza viruses are filamentous and can be up to 500 nm long [28]. 

As previously mentioned, influenza A viruses are divided into subtypes based on two proteins on the surface of the virus: hemagglutinin (HA) and neuraminidase (NA). Human influenza viruses are named according to their type, subtype, strain number, place, and year of isolation. According to their HA and NA antigenic properties, virus subtypes are named as a combination of 18 different HA subtypes and 11 different NA subtypes, such as HxNy [29,30]. Influenza viruses can differ in pathogenicity and virulence according to their HA:NA ratio, usually around 4:1 [28,31].

Among the different types of influenza, influenza A viruses (IAV) are the most pathogenic for humans. Thus, IAV are the main target for the development of seasonal vaccines to control morbidity and mortality in at-risk populations. IAV have a vast array of hosts including poultry, swine, horses, aquatic mammals, bats, cats, dogs, and birds. Adding to the broad range of species that IAV infect, through antigenic drift and antigenic shift, IAV can acquire the ability to surpass the species barrier. This can allow the emergence of new strains that become a threat to humans [4].

There are two strains of influenza B circulating, B/Victoria/2/1987 and B/Yamagata/16/1988, which arise according to geography. Unlike IAV, the type-B influenza virus is only susceptible to undergo mild, gradual antigenic drift. Influenza C is less dangerous because it lacks neuraminidases on its surface and has a different hemagglutinin, translating into fewer outbreaks and moderate symptoms [29,32].

**Figure 1:**
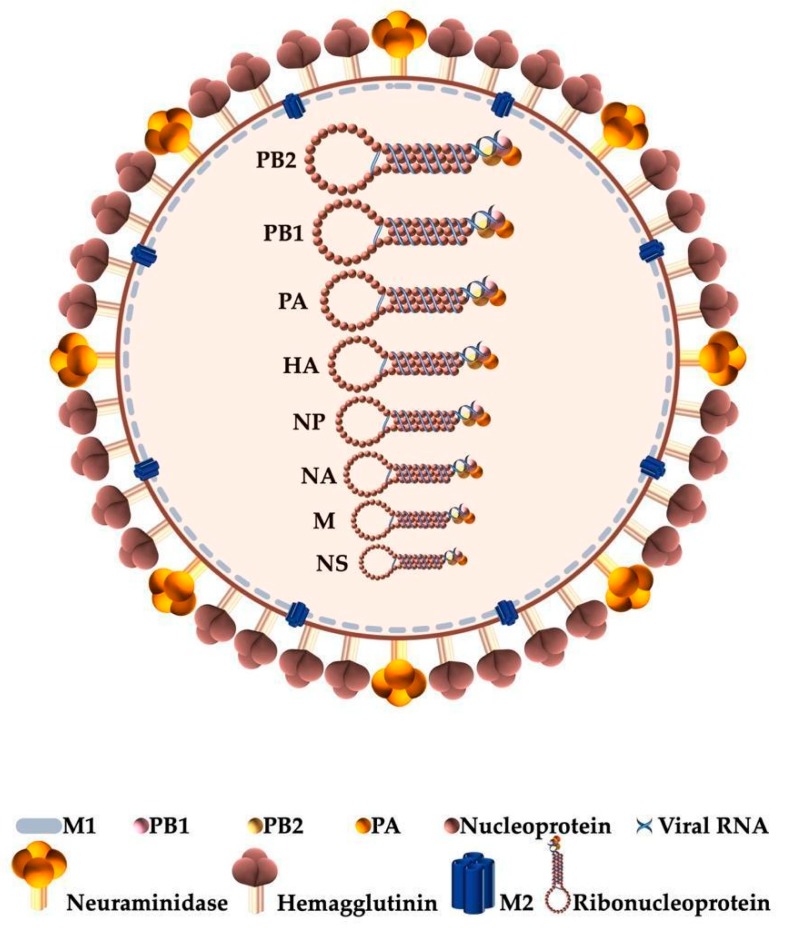
Schematic representation of the influenza virion. The viral genome is depicted highlighting the 8 single-stranded RNA segments (PB2, PB1, PA, HA, NP, NA, M, and NS) organized into ribonucleoproteins and here displayed by their decreasing size. Each ribonucleoprotein complex includes one trimeric viral RNA polymerase (PB1, PB2, and PA), in addition to nucleoproteins wrapped in viral RNA. (M1—viral matrix protein; PB1—polymerase basic protein 1; PB2—polymerase basic protein 2; PA—polymerase acidic protein; M2—ion channel).

Structurally, the influenza virion (Figure 1) has an envelope formed by a lipid bilayer embedded with the glycoproteins hemagglutinin (HA) and neuraminidase (NA) which allows the virus to bind to a host cell. M2 is a transmembrane protein that forms proton channels in the viral envelope [32]. The viral matrix protein M1 is membrane associated and forms a rigid matrix layer under the viral envelope. It mediates the encapsidation of RNA-nucleoprotein cores into the membrane envelope. Inside the virion particle, the viral proteins are organized into super-coiled viral ribonucleoproteins (vRNPs) comprising the eight, negative sense, single-stranded ribonucleic acids (RNAs) segments plus a trimeric assembly, the viral polymerase (VP), which includes the polymerase basic protein 1 (PB1), polymerase basic protein 2 (PB2), and polymerase acidic protein (PA). These vRNPs are the central elements in the virus life cycle which are complemented by the host cellular processes [33,34]. 

A general structure of the influenza virion is depicted in Figure 1. 

Table 1 summarizes the function and structure of each protein coded by the eight vRNA segments.

In addition to the proteins presented in Table 1, accessory proteins that support regulatory functions in the host or the virus can also be coded depending on the viral strains [38,39].

## 3. Non-Structural Protein 1

### 3.1. Structure and Function

**Figure 2:**
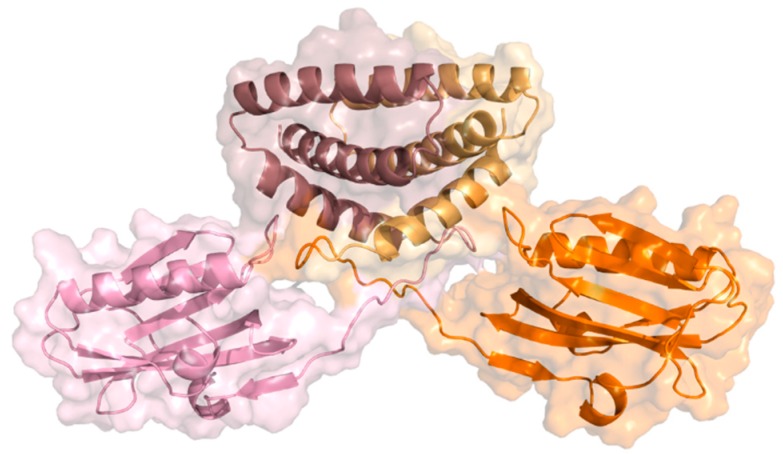
Ribbon and surface representations of the NS1 dimer highlighting its structural domains. Subunits 1 and 2 are represented in pink and orange, respectively; N-terminal RNA-binding domains (RBDs) are represented in dark pink and light orange (top) and effector domains (EDs) in light pink and bright orange (bottom) (PDB ID: 4OPH) [40].

NS1 is a highly conserved [40] and multifunctional protein with approximately 26 kDa molecular weight and 215 to 237 amino acids, resulting from the collinear transcription of the eighth vRNA segment. This viral protein has four different structural regions: two globular domains, a linker region, and a C-terminal disordered “tail”. The N-terminal RNA-binding domain (RBD) comprises the first 73 amino acid residues, the effector domain (ED) residue 85 onward until the end of the structured part of NS1, and the C-terminal tail (CTT) is a disordered region with about 20 residues. The linker region (LR), a short and flexible region with a variable number of residues, connects both globular domains [38,39]. NS1 can adopt different conformational states due to the high flexibility and variable composition of the linker region [41]. A schematic view of the structure of the NS1 dimer is depicted in Figure 2.

Both X-ray crystallography [42,43,44,45] and nuclear magnetic resonance (NMR) [46,47,48,49] were used to solve NS1’s structure, which is typically represented in its dimeric form [45,50]. RBD has two long antiparallel α-helices (from Asn4 to Asp24; and Pro31 to Leu50) linked by a short loop and a third helix (from Ile54 to Lys70) before the linker region [50]. To bind to double-stranded RNA (dsRNA), RBD dimerizes forming a group of six antiparallel α-helices. A pair of highly conserved Arg38 residues, one per dimer subunit, seem to play a critical role in the interaction with dsRNA, in addition to a charged residue, lysine, at position 41 (Lys41) [38,39]. Some authors propose that binding to dsRNA allows NS1 to assemble into an oligomeric tubular structure that protects the dsRNA molecule, and thus block cellular antiviral processes [41,43,51]. The ED has a seven-strand twisted β-sheet as its main structural element. The structure is comprised by an initial β-strand in the N-terminus followed by a short helical element, five main antiparallel β-strands, a α-helix across the β-sheet, and an additional β-strand before the C-terminus region [44,46]. 

**Figure 3:**
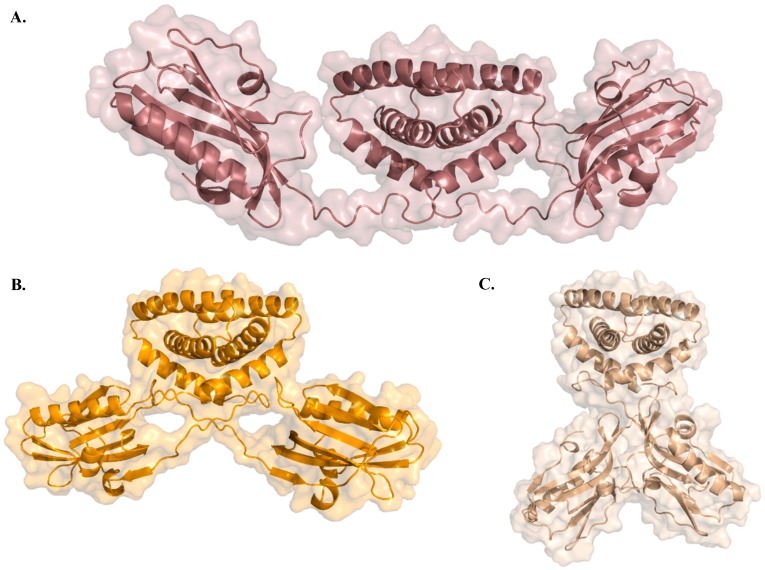
Three X-ray crystallography NS1 structures illustrating the different conformational arrangements of the NS1 dimer: (**A**) “Open” conformation (PDB ID: 6O01 [53]), (**B**) “semi-open” conformation (PDB ID: 4OPH [41]), and (**C**) “closed” conformation (PDB ID: 4OPA [41]).

In cells, full-length NS1 is likely a homodimer [52] with several conformational states as “open”, “semi-open”, and “closed” [41], depicted in Figure 3A–C, respectively. Previous studies have hypothesized that NS1 conformational states are strain dependent with variations being attributed to different linker sizes and residue composition [41]. However, more recently, evidence has been provided that even in the presence of a shorter linker, it is possible to observe open conformations [53]. Taken the available full-length structures of NS1, it has been proposed that NS1 can shift between different oligomeric conformational states, depending on the function being carried out. This could be related to cellular location, virus lifecycle, or even the presence of different molecular partners with whom NS1 interacts [53]. 

### 3.2. Post-Translational Modifications: Phosphorylation, SUMOylation, and ISGylation

Several post-translational modifications (PTMs) have been identified in NS1, in particular, phosphorylation, SUMOylation, and ISGylation [54]. NS1 features several phosphorylation sites, namely Thr215, Ser42, and Ser48. From a functional point of view, these PTMs remain to be fully understood. Although phosphorylation in general does not appear to greatly affect viral replication and it is strain dependent, Ser42 phosphorylation makes binding of dsRNA more difficult, and thereby decreases viral replication rates [55,56,57]. Recent studies showed that phosphorylation may also occur at Thr49, which inhibits NS1’s association to TRIM25 and decreases NS1 ability to interfere with the IFN cascade [58]. Another study showed that Thr80 phosphorylation reduces interaction with retinoic acid-inducible gene 1-like receptor dsRNA helicase enzyme (RIG-I) hence making NS1 unable to inhibit the IFN cascade [59]. Some H5N1 subtype strains display a mutation in Asp92 to Glu92 which reduces the strength of the hydrogen bonding interaction between Glu92 Ser195 and Thr197. These two residues (Ser195 and Thr197) are then available to further interact or be phosphorylated which translates into a more virulent strain [42]. Moreover, Thr215 can also be phosphorylated and, since, this is nuclear localization signal 2 (NLS2) region, it could lead to disruption of NS1 nuclear location [60].

SUMOylation is a post-translational modification by the SUMO (Small Ubiquitin-like Modifier) family of proteins which is involved in cellular processes like nuclear-cytosolic transport, transcriptional regulation, apoptosis, protein stability, response to stress, and progression through the cell cycle [61]. SUMOylation of NS1 by SUMO1 occurs in residues Lys219 and Lys221, both in the CTT, increasing NS1’s stability [55]. If both lysines are replaced by mutation, NS1 decays at a faster rate [62]. Some authors argue that SUMOylation happens not only by SUMO1 but also by SUMO2/3 and that the influenza viruses upregulate SUMOylation since it favors viral multiplication [63]. Lys219 and Lys70, which are conserved residues across viral strains, are crucial to abrogate the IFN signaling pathway associated with SUMOylated NS1. There are multiple potential sites for SUMOylation in NS1, but there seems to exist an intermediate optimal level of SUMOylation for maximal NS1 activity and pathogenic potential [63]. 

ISGylation, the conjugation of proteins with ISG15 (Interferon-stimulated gene 15 protein), has been demonstrated at several NS1 residues. The ISG15 conjugation system targets Lys41 at the nuclear localization signal 1 (NLS1) on the RBD, blocking NS1’s interaction with importin-α, critical to nuclear importation, hence decreasing the rate of viral replication [64]. Evidence suggests that Herc5 catalyzes this reaction, which can be especially damaging for NS1’s sequences that lack NLS2 in opposition to A/WSN/33 (H0N1) (WSN) viruses or more recent viruses (from 1989) [65]. This residue’s modification did not affect the dsRNA binding as its binding site does not superpose with the importin-α binding site [66]. Herc5-mediated ISGylation occurs on other lysine residues inhibiting different functions of NS1 and decreasing the likelihood of homodimer formation by a mechanism still unclear [67]. Influenza A viruses seem to counteract indirectly this effect since their function lowers the IFN levels, which consequently lowers ISG15 levels as well [68]. Influenza B viruses can overcome this through the action of E1-like ubiquitin-activating enzyme (Ube1L) that binds to ISG15 blocking it from accomplishing its function [68].

### 3.3. Function and Protein–Protein Interactions 

NS1 has a plethora of strategies to inhibit the host immune response due to its ability to establish multiple protein–protein and protein–RNA interactions. NS1 hampers different pathways both in the cytoplasm [69] and in the nucleus of infected cells. The presence of a nuclear export signal (NES) at the C-terminal end of the effector domain allows exiting from the nucleus to the cytoplasm. On the other hand, nuclear localization signals 1 and 2 (NLS1 and NLS2) direct NS1 to the nucleus. While NLS1 is part of the RNA-binding domain N-terminal region allowing NS1 to locate in the nucleoplasm, NLS2 is located in the C-terminal tail allowing NS1 to migrate to the nucleolus [70,71]. 

**Figure 4:**
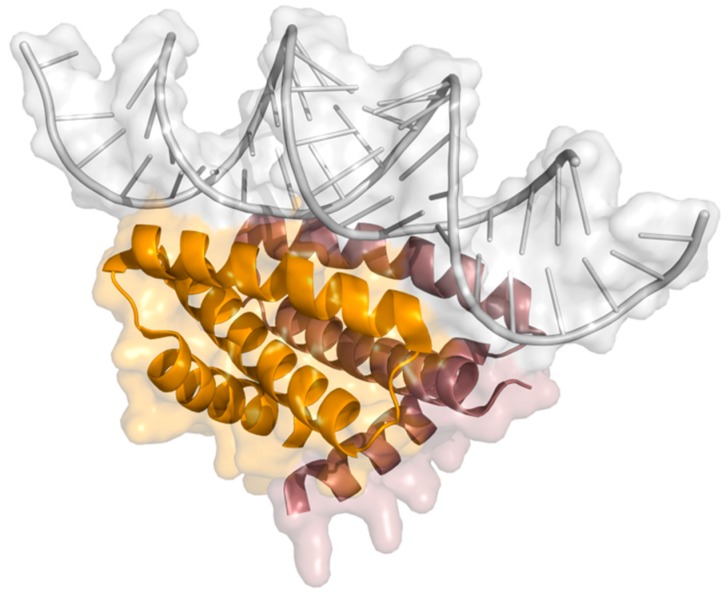
X-ray structure of RNA binding domain of NS1 bound to dsRNA (PDB ID: 2ZKO) [76] (orange, NS1-RBD monomer 1; pink, NS1-RBD monomer 2; gray, dsRNA).

Hindering of the IFN induction cell signaling is critical in influenza’s infection since production of cytokines like type I interferons is the hosts innate immune prime response to withhold the infection [55]. IFN induction starts by activation of several processes in a cascade and NS1 counteracts them in different ways. RIG-I is active after identification of viral dsRNA leading to ubiquitination of TRIM 25 (tripartite motif-containing protein 25) [72]. As a consequence of this event, RIG-I constitutes an important sensor of influenza’s infection [73]. RIG-I binds to NS1 in the same RBD highly conserved site comprising amino acids Arg38 and Lys41, where dsRNA also binds, as depicted in Figure 4 [74]. Although, RIG-I and NS1 form a complex observable by immunoprecipitation, their structural relationship remains unclear [47,72]. Jureka and coworkers recently presented results that show the importance of NS1 Arg21 in RIG-I’s activation and that its mutation to glutamine inhibits RIG-I binding. Nonetheless, this point mutation has no effect on TRIM25 nor dsRNA binding [75].

**Figure 5:**
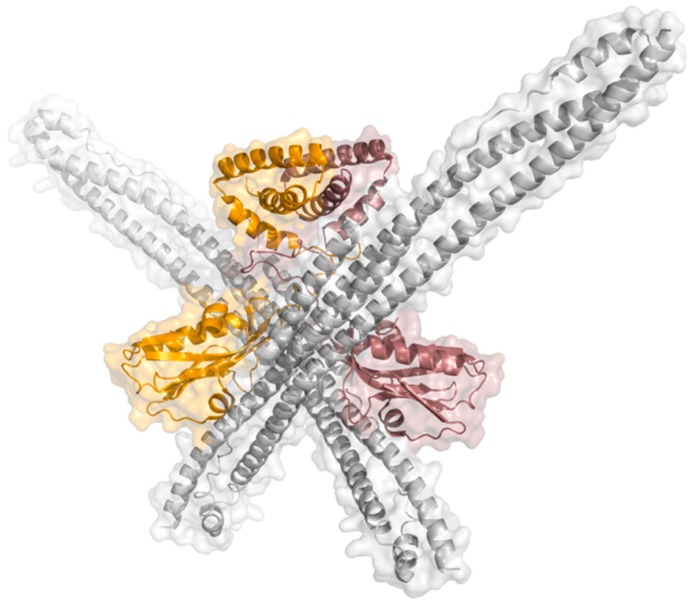
X-ray structure of NS1 dimer bound to TRIM25′s coiled-coil domain (PDB ID: 5NT2) [80] (orange, NS1 monomer 1; pink, NS1 monomer 2; gray, TRIM25′s coiled-coil domain).

TRIM25 [77], a ubiquitin ligase, and riplet [78] are essential for IFN production and promote RIG-I’s ubiquitination. NS1 is able to bind to both these proteins, enhancing its IFN’s inhibitory action. NS1 binds to TRIM25 (Figure 5) preventing the RIG-I’s ubiquitination [79]. Structural studies by Koliopoulos and coworkers also show that NS1 oligomers can cross-link different molecules of TRIM25 [80].

Recently, Wang and collaborators identified two residues essential for NS1’s contribution to viral pathogenicity and its cell concentration. Mutation of Ser212 to proline in avian H7N9 decreases the NS1 ability to stop the host’s IFN response and to enhance viral replication. Likewise, mutation of Ile178 to valine prompted NS1 degradation through the proteasome pathway [81].

**Figure 6:**
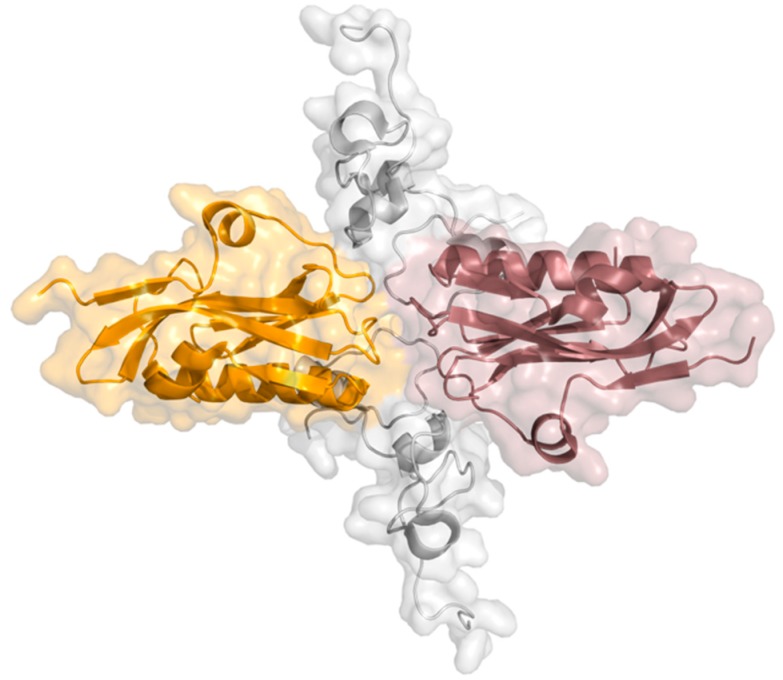
X-ray structure of NS1 effector domain bound to CPSF30′s F2F3 finger (PDB ID: 2RHK) [87] (orange, NS1-ED monomer 1; pink, NS1-ED monomer 2; gray, CPSF30′s F2F3 finger).

Additionally, NS1 also attenuates the expression of host genes by interfering with the pre-mRNA processing machinery [82]. NS1 binds CPSF30 preventing polyadenylation of the host’s IFN pre-mRNA, leading to its accumulation in the nucleus and inhibiting expression of the IFN genes or IFN-related genes [83]. The polyadenylation of the viral mRNAs is not affected since it relies on the viral polymerase [84]. Binding to CPSF30 (Figure 6) also enables inhibition of mRNA transport from the nucleus to the cytoplasm and mRNA splicing [85]. It was suggested that the build-up of pre-mRNA in the nucleus is a source for cap snatching by the viral polymerase [86]. NS1 has a globally hydrophobic pocket comprising residues Lys110, Ile117, Ile119, Gln121, Val180, Gly183, Gly184 and Trp187, where NS1 binds to two (F2 and F3) of the five zinc fingers of CPSF30 [40]. Outside of this binding pocket, two residues, Phe103 and Met106, maintain the stability of the interaction [87]. As a consequence of the binding to CPSF30, NS1’s Trp187 is inaccessible, blocking NS1’s ED-ED dimerization [60]. Residues in the CTT mediate PABPII binding [88], preventing poly-adenine tail extension of mRNAs, and thus preventing nuclear export [89]. Other nuclear mRNA export factors, such as NXF1, p15, Rae1, E1B-AP5, and Nup98, have also been reported to bind to NS1 [90,91]. Taking into account all these interactions, it becomes clear the relevance of NS1 in inhibiting the antiviral response of the host cell, as well as, the interference on the effective communication between neighboring cells. 

NS1 inhibits double-stranded RNA-activated protein kinase (PKR), 2′,5′-oligoadenylate synthetase (OAS), ribonuclease L (RNAse L), and phosphoinositide 3-kinase (PI3K) of the host cell, interfering with multiple cellular pathways. PKR is a kinase from the eukaryotic initiation factor 2α (eIF2α) that, after activation by dsRNAs, inhibits translation, a critical aspect harmful to viral growth. In previous studies, the interface for the NS1-PKR binding was thought to comprise residues Ile123 and Asn127 [92] which would block PKR’s conformational change needed for dsRNA binding [93]. Recent studies have shown that NS1 residues Arg35 and Arg36 are involved in the direct binding of NS1 and PKR, blocking PKR’s activation and enhancing the influenza’s virulence [94]. The affinity of NS1 to dsRNA is high enough to overcome in vivo competition with OAS. This means that NS1 binds preferentially to dsRNA instead of binding to OAS. This could indicate that the primary function of dsRNA binding is to make it unavailable to OAS, hence, stopping the IFN cascade by the activation of RNAse L through OAS [95].

To enhance pro viral responses, viruses have different strategies such as delaying apoptosis through PI3K activation, an important checkpoint for cellular homeostasis [96]. NS1 interacts with the SH_2_ domains of the p85β subunit of PI3K via residues Tyr89 and Pro164. It has been suggested that the dimerization of the ED is not necessary for the interaction with PI3K [97]. Recently, additional NS1 interactions have been proposed, such as the one with nucleolar RNA helicase 2 (DDX21). This protein inhibits viral polymerase (VP) aggregation by PB1 viral subunit binding. Due to NS1’s binding to DDX21, VP is then able to assemble all the subunits (PA, PB1 and PB2) and continue to catalyze viral RNA synthesis [98]. NS1 also binds to the 5′UTR of viral mRNAs, translation initiation factor 4G1 (elF4G1) (amino acids 81 to 113) [99], and polyadenine binding protein I (PABPI) [100] forming a heterotrimeric complex that inhibits viral translation. Cytoplasmic polysomes were found to contain NS1 and hStaufen, a protein involved in the transport of mRNAs to the active translation sites [101]. Zhu, Zheng, and collaborators had recently unveiled the interaction of NS1 with the nucleolar and coiled-body phosphoprotein (NOLC1). The authors observed that, after binding, there was an increase in apoptosis of host cells, which seems counterintuitive considering the role of NS1 in the viral cycle, and thus awaits future clarification [102].

For a more holistic understanding of the multitude of virus–host interactions, data intensive approaches such as interactomics will play a role. Shapira and coworkers [103] found that NS1 promotes up-regulation of 45 virus-specific regulated genes (VSRGs) from which 24 have an important role in viral replication or the host’s IFN response. DeChassey and coworkers [104] got an extensive interactome of both NS1 and NS2, identifying more than 560 interactions and 33 proteins that interacted only with NS1. These intricate interaction network maps help to delineate novel strategies to use NS1 as a potential therapeutic target. Raman and coworkers [105] developed an interactome of NS1 in pigs which could be evaluated for human applicability. They identified 192 proteins (92 already documented), some were new interactors like heterogeneous ribonucleoprotein particles (hnRNPs) C, K and U, interleukin enhancer-binding factor 3 (ILF3), and ATP-dependent RNA helicase (DDX1), while others like PABPI, DEAx box helicase 9 (DHX9), DDX21, and Crk-like protein L (CrkL) got confirmation in this study. Recently, another interactome analysis of NS1 showed that, in addition to all the PPIs already described, NS1 is involved in the splicing the pre-mRNA through binding to the pre-mRNA-processing factor 19 (PRP19). This new interaction should be further studied in order to assess its relevance for viral pathogenicity, since the same study showed that cells without endogenous PRP19 also showed decreased IAV replication [106].

NS1 has gained motifs in its CTT which have conferred to NS1 the ability to interact with a wider collection of proteins [107]. There is evidence in the literature that NS1 may interact with the following additional partners: human PAF1 transcription elongation complex (hPAF1C); parafibromin (CDC73), global transcription activator (SNF2L) [108]; chromodomain-helicase-DNA-binding protein 1 (CHD1) [109]; WD repeat-containing protein-5 (WDR5) [109]; β-tubulin [110]; nucleolin, fibrillarin, and nuclear B23 [70]; RNA-associated protein 55 (RAP55) [111]; RNA binding protein nuclear factor 90 (NF90) [112]; heat shock protein 90 (HSP90) [113]; NOD-like receptor family, pyrin domain containing 3 protein (NLRP3) [114]; inhibitor of nuclear factor kappa-B kinase subunits α and β (IKKα and IKKβ) [115]; poly(a)-binding protein II (PABII) [89]; human guanylate-binding protein 1 (hGBP1) [116]; transcription factors p53 and cleavage and polyadenylation specificity factor subunit 4 (CPSF4) [117]; PDZ and LIM domain protein 2 (PDlim2) [118]; membrane-associated guanylate kinase inverted 1, 2 and 3 (MAGI-1,2 and 3, respectively), scribble and discs large homolog 1 (Dlg-1) [119,120]; tyrosine-protein kinase Src (c-Src), tyrosine-protein phosphatase non-receptor type 1 (PTPL1) and reversion-induced LIM protein (RIL) [121]; and adapter molecule crk (CrkI and CrkII) [107].

**Figure 7:**
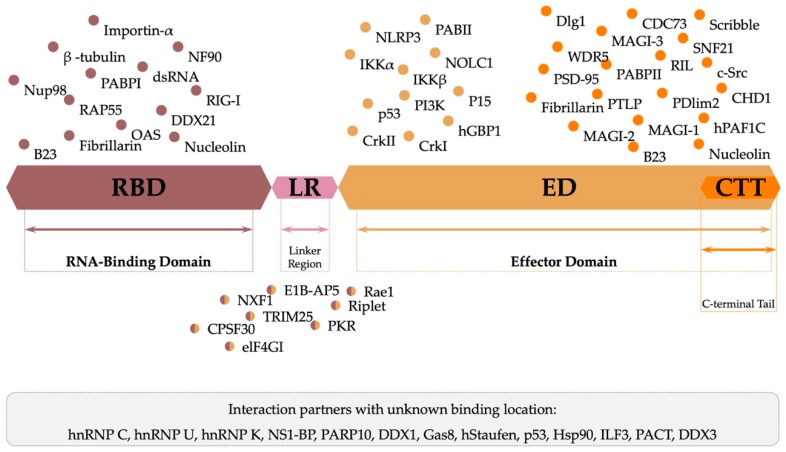
Graphical representation of known NS1 protein-protein and protein-RNA interactions and their respective site of interaction. (all proteins and interactions mentioned in the figure are referenced in the text).

It remains to be seen, among the multitude of interactions reported (Figure 7), which are relevant in vivo and at what stages of the virus life cycle. But the general evidence coming out of all these interactions mediated by NS1 points to pathways that enhance viral replication, while maintaining the host’s immune response weakened.

### 3.4. NS1: Target to Manage Influenza Outbreaks

#### 3.4.1. NS1-Modified Virus as Vaccines

NS1 has been related to increased pathogenicity and virulence of influenza. Viruses that fail to express NS1 may cause less severe symptoms and show decreased ability to proliferate. For example, the IFN cascade is not as affected and will perform normally under lack of NS1 expression in the host’s cells. NS gene truncations or deletions translate into attenuated viruses [122] as confirmed by a H1N1 influenza strain which lacked the NS1 reading frame (ΔNS1-H1N1) and was tested successfully as an intranasal vaccine in Phase I clinical trials with healthy adult volunteers [123]. Following this study, serum and nasal wash analysis for IgA revealed that it can neutralize influenza’s viruses not only from the subtype H1N1, but also from H3N2 and H5N1 subtypes. IgG, on the other hand, is only effective on the subtype tested, H1N1 [124].

A different study used a delNS1-H5N1 as a live attenuated vaccine virus. After an intranasal monovalent dose, results revealed its safety and tolerability. Moreover, 75% of the participants displayed seroconversion after one immunization and IgA serum levels were increased after a second immunization [125].

In addition to the abovementioned studies, a trivalent vaccine succeeded in Phase I/II trials. An intranasal vaccine formulation was used for a delNS1-trivalent vaccine comprising the strains H1N1, H3N2 from influenza A viruses and a type-B influenza strain. Similarly to other clinical trials, this vaccine was also safe and well tolerated and IgA levels were also increased as expected [126].

#### 3.4.2. Experimental and In Silico Approaches Towards a New Therapeutic Path

Vaccination is the first approach to prevent influenza episodes, but prevention only goes so far, as explained previously. The second most used method against influenza outbreaks is the use of antiviral drugs. M2 inhibitors, known as adamantanes (amantadine and rimantadine) have limitations due to increased resistance among the IAV. Although some strains are also becoming resistant to the most used neuraminidase inhibitors (oseltamivir, zanamivir, and paramivir), these are still prescribed in critical infection cases, high-risk groups, or hospitalized patients [23].

With the high viral mutation rates and resistance-related problems associated with the commonly used therapeutic targets, the key to the development of new antivirals is the identification of novel therapeutic targets [127]. In this context, NS1 presents itself as a promising target due to its multifunctional nature and central role in virus biology and infection mechanisms. Thus, it is key to pinpoint all the relevant interactions and ligand-binding sites in NS1 to enable the discovery of small molecules with the goal of developing new therapies [24]. One of the most relevant characteristics of NS1 to be considered is its ability to inhibit the innate immune response of the host, restricting the cascade response of IFN at different levels and increasing the level of virulence of the influenza virus [128]. One way to decrease NS1 expression is through the use of small interfering RNAs (siRNAs). This approach strictly marks viral genes with zero impact on host pathways and expression, which should decrease undesirable side effects and toxic effects [129]. Wu and coworkers synthesized three antisense RNA oligonucleotides targeting the NS gene, and each one worked individually well *in vitro*. However, in vivo the results were not very promising. The authors recognized that the results were improved when the three sequences were used simultaneously. Rajput and coworkers [130] tried to mute NS genes post-transcriptionally by administering wild type (WT) siRNAs *in vivo*. Their results showed that siRNAs could strongly inhibit viral replication, justifying this as a valuable approach applicable to antiviral drugs that aim to inhibit NS1 indirectly.

Some proteins and peptides, in particular antibodies (Abs), were found to effectively and specifically inhibit NS1’s activity. The monoclonal antibody (mAb) D9 was able to interact with an avian influenza virus’ epitope of NS1, although not inhibiting it [131]. Still, regarding avian influenza, several other mAbs were found to bind to virus’ proteins, in particular, NS1 [132]. In a different study, mAb 2H6 was found to bind to NS1, inhibiting the interaction between NS1 and double-stranded RNA [112]. Avian influenza was also tested with 11 mAbs, four of them anti-NS1 mAbs which showed reactivity against recombinant NS1 [133].

Rational discovery and design of new lead compounds for therapy development is significantly helped by the knowledge of the high-resolution three-dimensional structure of the therapeutic target. In the case of NS1, several structures of the full-length protein and its functional domains (RBD and ED) are available at the Protein Data Bank (PDB) [134]. Among the 44 NS1 structures available to date (February 2020), several are in complex with different molecular partners—dsRNA or other proteins. This multitude of examples helps in the identification of NS1 main interaction sites and thus in the discovery and design of inhibitors of these interactions. Both experimental and in silico methods may be used to identify suitable ligands for NS1 by blocking NS1 activity or preventing some key interaction, and thus allowing the identification of potential lead compounds and drug candidates [135].

Regarding drug design, NS1 is a target to be considered due to its involvement in multiple essential pathways in influenza’s biology [135]. NS1 was initially identified as a viable target through yeast-based screening assays with 2000 compounds. Of these, four compounds emerged capable of inhibiting NS1 activity. Initially, Basu and coworkers [136] conducted a study aiming at identifying compounds able to block NS1 activity without having repercussions on the hosts’ proliferation. As NS1 is toxic to yeast, the NS1 inhibitor is as successful as the yeast cells regain their normal growth [137]. It was found that three compounds (NSC128164, NSC109834, and NSC95676) decreased the levels of NS1-specific viral RNAs. Another compound (NSC125044), although not interfering with the viral RNAs levels, increased IFN’s induction. These four compounds seem to act on two different antiviral mechanisms. This yeast-based assay showed that it is possible to target accurately NS1 and that the action on NS1 decreased the virus load. The action of these compounds is restricted to specific interactions with influenza’s NS1, as activity in other viruses has not been inhibited. These results proved that NS1 may be a target for small molecule inhibitors with clinical applicability. Walkiewicz and coworkers [138] pursued one of these four compounds to further investigate how it modulates NS1’s action, optimizing the prior hits. One of these compounds—JJ3297—was shown to inhibit viral replication by shifting the IFN signaling cascade into regaining its normal function. This is important if the infection is in its early stages because JJ3297 is effective in restoring the IFN cascade, preventing the spread of the infection to neighboring cells. However, the compound is less effective if the infection has already spread to a high cell number. The mechanism of action of JJ3297 remains unclear, but it seems to be related to the essential presence of RNAse L.

In 2012, another compound—NSC125044—was tested by Jablonski and coworkers [139] for activity evaluation of the molecule’s core after stripping it of accessory regions, and a structure-activity relationship (SAR) was established. Using a live cell virus replication assay, the authors concluded that the best SAR for this binding site was represented by a chemical entity with a large hydrophobic core with a weakly basic function on its left-hand side, whereas the right-hand side should have an aromatic ring linked by an amide bond before a short methylene chain. In this study, A9 and A22, small molecules that are derivatives of NSC125044, were the most promising molecules, A22 being 10 times more potent than A9. Recently, Kleinpeter and coworkers [140] continued Jablonski’s research [139]. Both molecules bind to NS1-ED and, by mapping the interaction region through X-ray crystallography, it was shown that they share the same binding pocket as CPSF30, meaning that these compounds can act as inhibitors of the NS1-CPSF30 interaction.

In addition, the ML303 family of compounds was passed onto in vivo studies carried out by Patnaik and coworkers [141]. This pyrazolopyridine family of compounds is a group of NS1 antagonists and exhibited rehabilitation of the IFN cascade, previously inhibited by NS1, and a remarkable antiviral activity in-cell culture. Another study using yeast-based assays was conducted by Kong and coworkers [142] targeting NS1’s interaction with CPSF30. They tested Chinese medicine substances to assess if they could inhibit this interaction site. The most promising results came from an oral liquid substance called “Shuanghualian”.

Other authors performed high-throughput screening (HTS) assays, using fluorescence polarization (FP) [143,144,145] and radiolabeled RNA [146], to identify inhibitors of NS1’s interaction with dsRNA. Mata and coworkers [143] studied a naphthalimide family of compounds to identify if they could revert the function of NS1 in blocking the replication machinery of the host. They concluded that these compounds could be used as upregulators of hypoxia-inducible factor-1 (REDD1), a mammalian target of rapamycin complex 1 (mTORC1) inhibitor, which is a defense factor [147]. Cho and coworkers [144] carried out a FP-based screening study that identified six promising molecules as NS1 inhibitors. Epigallocatechin gallate (EGCG) was selected as the most effective using a minimal dosage. They proposed that EGCG interferes with the binding of dsRNA to NS1 because it interacts with Arg38, which is essential for dsRNA binding. You and coworkers [145] also used FP-based assays to test a family of quinoxalines, which share a bicyclic ring with the ability to incorporate polar side chains. They conducted a SAR study concluding that two compounds were effective at binding to the NS1 dsRNA binding domain, inhibiting the binding of dsRNA. Martínez-Gil and coworkers [148] tried a different approach. After high-throughput screening, compound C3 came up as a potential candidate against influenza’s infections since its effect of increasing IFN levels was not affected by NS1, apparently because it activated a different pathway or because it acted downstream of NS1’s targets. This molecule seems promising as a broad-spectrum antiviral since its action is not NS1 dependent, but, instead, IFN inductive. Maroto and coworkers [146] used radiolabeled RNA to check if the NS1’s binding site to vRNA could be a new antiviral drug target and, if so, to identify some contenders. They analyzed 27,520 compounds and three of them could decrease NS1-vRNA binding proving that this could be a target for new drugs.

Virtual screenings have also helped in the discovery of hit compounds. CPSF30 is involved in direct pre-mRNA processing and has its activity altered due to NS1 [149]. On the basis of this assumption, a study was conducted using molecular docking and molecular dynamics, starting with more than 30,000 initial compounds [150]. This study allowed the selection of two compounds capable of inhibiting the activity of NS1 related to CPSF30. Another study performed a docking-based screening of 200,000 compounds with AUTODOCK [151]. The authors picked two compounds to proceed out of 17 hits with positive outcome. These compounds displayed the ability to inhibit the activation of an antiviral pathway by interfering with NS1’s interaction with dsRNA [152].

Although NS1 establishes interactions with multiple molecular partners, the interaction between NS1 and dsRNA is one of the most explored. Molecular dynamics (MD) simulations and free energy calculations have been used to understand the molecular basis of dsRNA recognition by NS1 [153]. Free energy calculations can be performed by combining molecular mechanics with Poisson−Boltzmann surface area (MM/PBSA) or generalized Born surface area (MM/GBSA) calculations. This approach allows the estimation of ligand-binding affinities by combining molecular mechanics and continuum solvation models [154]. MD simulations were also used to explore potential interactions of NS1 with membrane proteins [155], an approach that might help in the understanding of the overall process involving the virus and virion interactions with the host cell [156]. Table 2 summarizes the main aspects of the studies mentioned above.

#### 3.4.3. NS1’s Role in Influenza’s Host Tropism

Host tropism refers to the specificity of some pathogens to infect particular hosts or host tissues. Molecular and biological characteristics of the host organism determine host tropism, i.e., the ability of a virus to interact with a particular characteristic of a host. In the case of influenza virus, quantitative analysis of surface interacting molecules and immunohistochemistry assays stand central to the understanding of host tropism, although other approaches should not be neglected [160]. Computational approaches like data mining and machine learning (ML) have been used. For example, random forests (RF) was used to build 11 computational models from sequences of proteins isolated from influenza, producing highly accurate prediction models capable of determining the host tropism of individual influenza proteins. In this study, NS1 was predicted to have a large number of interactions with different hosts (high host tropism) [161].

Influenza viruses, in particular, have a high risk of being transmitted through animal carriers (zoonotic risk [162]). The distinction in host tropism protein signatures can be used to train ML prediction models for zoonotic strain prediction. Using a dataset consisting of 13,998 strains of the influenza A virus from the Influenza Research Database [163], after removing invalid sequences of the 11 proteins, NS1 included, host tropism signature features were transformed from the protein sequence to yield 146 feature vectors comprising several amino acid-based characteristics. This dataset was then used to train an RF prediction model, i.e., an ensemble of decision trees trained with randomly selected features and instances that were then combined with a bagging algorithm to maximize the value of the information on the dataset. This model can predict the zoonotic classification of the virus (in this case, avian, human, or zoonotic), and thus could be eventually used as a predictive tool with social, economic and public health impact [161].

Due to their ability to evade species barriers influenza strains have been detected in a larger number of different host’s species [164]. Thus, tackling influenza infections and pandemics requires the knowledge of the host range of different virus strains. In a recent study, a multi-host dataset of 674 different influenza strains organized into three host categories (avian, human, and swine) was compiled. The authors multi-labelled the sequences depending on the host’s species they were isolated from and their parent-strains. For example, if a human isolated virus reassortment occurred from both avian and swine parent-strains, then, the new strain would be labelled as avian, swine and human. This dataset ended up with seven different classifications: avian, human, swine, human-swine, human-avian, avian-swine, and human-avian-swine. This classification enabled the representation of the host range for each sequence through multi-label learning, i.e., the possibility of each sequence to belong to more than one individual class. Finally, the dataset was trained using different ML models, such as decision trees, and it was possible to pinpoint the most discriminative and combinatorial positions, regarding the host range. NS1 was one of the proteins associated with high human host range [165].

## 4. Conclusions

The morbidity and mortality associated with seasonal flu epidemics have a significant social and economic impact in contemporary societies, being from the large number of missed working days per year, or even more significantly from the increase in mortality of senior citizens and other high-risk groups. Moreover, the permanent and latent menace of a flu pandemic threatens the safety and way of life in our societies. The inefficient coverage provided by flu vaccines and the rapid development of resistance against the known antivirals by many influenza strains, highlights the need for new antivirals directed against novel biological targets.

Non-structural protein 1 (NS1), the subject of this review, plays a key role in influenza virus biology and mechanisms of infection through a multitude of molecular interaction partners, host cell activities, and structural plasticity or polymorphism. These multiple functions are mediated by multiple molecular interactions and various binding spots or pockets that could be targeted to develop novel antiviral therapies. NS1-directed antivirals could eventually be used in monotherapy or as part of combination therapies allowing synergistic effects against resistant strains [166].

In addition to the knowledge on the molecular mechanisms of action of influenza proteins, the characterization of host tropism of influenza virus needs also to be addressed with the aim of predicting the viral strains with higher probability of successful zoonotic transmission. Regarding host tropism, the importance of NS1 has also been highlighted and shown to directly influence the host range through its action on interferon response [161,167].

Progresses in the structural and functional characterization of influenza’s NS1 should pave the way for a more holistic understanding of this protein, and thus chart new paths for more efficacious ways of combating influenza outbreaks. Lead and tool compounds directed to NS1 have been identified, allowing validation of the protein as a potential therapeutic target. The coming years will see the discovery of new compounds and eventually the progress to the clinical setting of inhibitors of influenza’s NS1.

## Figures and Tables

**Table 1:** Summary of the functions and interactions of the proteins coded by each RNA segment of influenza viruses (aas, amino acids).

RNA Segment	Protein(s) Coded	Function [20,31,35]	Structural Data [20,31,35,36]
**1**	**PB2** 759 aas	Located in the nucleus of infected cells; Signals the viral polymerase passage to the host’s nucleus; Enhances the formation of the cap structures necessary for viral messenger RNA (mRNA) transcription; Located in the mitochondria of infected cells [37]; Inhibits Interferon-β; Helps determine host range.	The three proteins, PB2 (polymerase basic protein 2), PB1 (polymerase basic protein 1) and PA (polymerase acidic protein), form the viral RNA polymerase, responsible for viral RNA transcription and replication.
**2**	**PB1** 757 aas	Responsible for the elongation of the primed nascent viral mRNA; Located in the nucleus of infected cells; Enhances the association of the 3 subunits of the RNA polymerase complex.	
**3**	**PA** 716 aas	Functions still unknown, but evidence points to helicase-like functions; Important for viral transcription; Assembly of the polymerase complex.	
**4**	**HA** 550 aas	Attaches the virions to the sialic acid (SA) moieties of the host’s receptors; Around 30% variation between subtypes.	Hemagglutinin (HA) is a homotrimeric integral cylinder-like membrane glycoprotein on the virus surface; 4 antigenic sites with direct impact on virulence and pathogenicity of the virus.
**5**	**NP** 498 aas	Binds non-specifically to single-stranded RNA (ssRNA); Encapsidates viral RNA; Helps recruiting RNA polymerase for synthesis of viral positive-sense RNA (cRNA); Related to host range.	Nucleoprotein (NP) is a 56 kDa basic protein; RNA-binding protein; Structural unit of RNPs; Forms oligomers stabilized by vRNA.
**6**	**NA** 470 aas	Unnecessary for virus replication; Required for budding of newly formed viral particles from surface of infected cells; Facilitates virus movement to the target cell by cleavage of sialic acids from respiratory tract mucins; Helps the release of virions from infected cells.	Neuraminidase (NA) is a homotetrameric integral membrane glycoprotein with 4 structural domains; Antigenic sites help circumvent the immune responses aiding on the virulence and pathogenicity of the virus.
**7**	**M1** 252 aas	Membrane-binding and RNA-binding protein; Forms a coat inside the viral envelope; Determines virion’s shape; Interacts with vRNP and other cytoplasmic domains of integral membrane proteins; Increases vRNPs export and decreases import; Helps assembly and budding of virions.	Matrix protein (M1) formed by a globular N-terminal domain and a flexible C-terminal tail; Oligomerization state and binding to lipid bilayer are highly dependent on pH.
	**M2** 97 aas	Vital for viral replication; Forms proton channel in virus envelope; Lowers the pH inside the viral particle to promote uncoating of RNPs; Modulates Golgi’s pH; Helps to stabilize HA’s native conformation during virus assembly.	Matrix-2 protein (M2) is a 97-residue single-pass membrane protein; Three segments: N-terminal outward segment, transmembrane (TM) helix, and C-terminal inward segment; TM helices from 4 subunits pack to form proton-channel; Highly conserved His37 and Trp41 residues.
**8**	**NS1** 230 aas	NS1 acts as a promoter of viral replication and an inhibitor of the host’s immune response; Present in the cytoplasm and nucleus of the host cell.	Non-structural protein 1 (NS1) has two structural domains—RNA-binding domain (RBD) and the effector domain (ED)—connected by a short linker (LR), and a disordered C-terminal tail (CTT).
	**NEP/NS2** 121 aas	Promotes viral RNA replication; Regulates vRNP’s export from the nucleus to the cytoplasm; RNA nuclear export; Interacts with the viral matrix M1 protein.	Nuclear Export Protein (NEP) has a protease-sensitive N-terminal domain (residues 1–53) and a protease-resistant C-terminal domain (residues 54–121) mostly formed by a helical hairpin.

**Table 2:** Summary of studies exploring influenza’s NS1 as potential therapeutic target.

Ligand	Activity	Method	Mode of Action	Ref
NSC128164 NSC109834 NSC95676	Blocks NS1’s action on IFN-related pathways	Yeast-based assay	Attenuates NS1 expression	[136]
NSC125044		Yeast-based assay	Induction of IFN-β and decrease of viral proteins expression	
JJ3297	NS1 antagonist	Yeast-based assay	Restores IFN antiviral state and inhibits virus replication	[138]
ML303	NS1 antagonist	Yeast-based assay	Restores IFN signaling pathway	[141]
“Shuanghualian”	NS1 antagonist	Yeast-based assay	Inhibits CPSF30 function by binding to NS1A binding site	[142]
Naphthalimide family of compounds	NS1 antagonist	Fluorescence polarization-based high-throughput assay	Up-regulation of REDD1 and mTOR1 inhibition	[143]
A9 (JJ3297) and A22	NS1 antagonist	Molecular docking, NMR, X-ray crystallography	Inhibit CPSF30 function by binding to NS1A binding site	[140]
30256 and 31674 (see paper)	NS1 inhibitor	Molecular Docking, Molecular Dynamics, MM-PBSA	Inhibit CPSF30 function by binding to NS1A binding site	[151,157,158]
(no name, see paper)	Blocks NS1	Molecular Docking	Inhibits dsRNA binding	[151,152]
Epigallocatechin gallate (EGCG)	Proposed binding to NS1’s Arg38	Fluorescence polarization-based high-throughput assay	Inhibits dsRNA binding	[144]
Quinoxaline family of compounds	NS1 inhibitor	Fluorescence polarization-based high-throughput assay	Inhibit dsRNA binding	[145]
(no name, see paper for three compounds)	Block NS1	Radiolabeled RNA	Inhibit NS1 binding to RNA	[146]
C3	Counteracts IFN blockage by NS1	Caspase 3-based high-throughput assay	IFN-induction	[148]
mAb	Binds selectively to NS1 of avian influenza virus	Antigen screening using recombinant peptides	Recognition of NS1’s presence	[132]
mAb	Binds to Thr49 region of NS1 of avian influenza virus	Enzyme-Linked Immunosorbent Assay (ELISA), surface plasmon resonance	Interferes with the virus ability to replicate	[159]
mAb D9	Binds to linear epitope in NS1	Western blot assay	Recognition of NS1’s presence	[131]
